# Field-Validated UAV-Based Deep Learning Framework for Automated Inspection of Power Transmission and Distribution Infrastructure

**DOI:** 10.3390/s26144478

**Published:** 2026-07-14

**Authors:** Gabriel Miguel Castro Martins, Murillo Ferreira dos Santos, Mathaus Ferreira da Silva, Juliano Emir Nunes Masson, Pedro Mendes Rocha Alves, Gabriela Ribeiro Cabral Chain

**Affiliations:** 1 Master Degree Program in Automation and Systems (PPGAS), Federal Center of Technological Education of Minas Gerais (CEFET-MG), Leopoldina 36700-001, Brazil; gabrielmcastro@outlook.com.br; 2 Robotictech Technology Services, Juiz de Fora 36036-330, Brazil; mathaus.silva@robotictech.com.br (M.F.d.S.); juliano.masson@robotictech.com.br (J.E.N.M.); pedroalves.xp@gmail.com (P.M.R.A.); 3 Elera Renewable Energies, Rio de Janeiro 22775-028, Brazil; gabriela.cabral@elera.com

**Keywords:** UAVs, deep learning, power transmission and distribution infrastructure, condition monitoring, asset inspection

## Abstract

The reliable inspection of power transmission and distribution infrastructure is essential for ensuring energy security, operational continuity, and asset reliability. Conventional inspection procedures are labor-intensive, costly, and often expose maintenance teams to hazardous environments. In this context, Unmanned Aerial Vehicles (UAVs) combined with artificial intelligence have emerged as an effective solution for large-scale infrastructure monitoring. This paper presents a field-validated framework for automated inspection of power transmission and distribution assets using autonomous UAV image acquisition and deep learning analysis. The proposed approach enables multiclass detection of electrical components and anomalies in high-resolution aerial imagery, without requiring computationally intensive 3D reconstruction. The framework integrates autonomous data collection, object detection, and dedicated condition assessment models into a scalable inspection workflow. The system was validated across six transmission and distribution lines located in five Brazilian states, covering 2925 support structures and a wide range of environmental and operational conditions. Experimental results achieved an overall mAP_50_ of 0.9572 across seven target classes, with individual scores ranging from 0.8945 for corrosion detection to 0.9935 for ceramic disc insulators. Complementary classification models achieved accuracies of 0.97 for insulator contamination assessment, 0.92 for pin attachment configuration, and 0.98 for ceramic pin integrity evaluation. The results demonstrate the feasibility of deploying Artificial Intelligence (AI)-assisted UAV inspections in real utility scenarios, providing a scalable alternative for preventive maintenance, asset management, and condition-based monitoring of electrical infrastructure.

## 1. Introduction

The reliable operation of power transmission lines is essential to modern energy infrastructure, requiring efficient inspection and maintenance of critical components such as crossarms, insulators, and bolts, as well as the detection of foreign objects like bird nests. Traditional manual inspection methods are labor-intensive, time-consuming, and often limited by human error and safety risks, especially given the vast scale and challenging environments of transmission networks. The integration of UAVs and photogrammetry has enabled the collection of high-resolution aerial imagery, providing a rich data source for automated analysis and asset management [1,2,3].

Despite these advances, several challenges persist. The diversity of component types, the small size of certain defects (e.g., bolt faults), and the presence of cluttered or variable backgrounds can hinder detection accuracy. To address these issues, researchers have developed specialized datasets, data augmentation techniques, and advanced model architectures that incorporate attention mechanisms, multiscale feature fusion, and transfer learning [1,4,5,6,7,8]. These innovations have improved the detection of small or occluded targets and enhanced model robustness across different inspection scenarios.

However, most existing studies focus on isolated components, limited datasets, or controlled experimental conditions, with relatively few works demonstrating large-scale deployment and validation under heterogeneous real-world operating environments. Ensuring robust performance across distinct geographic regions, environmental conditions, and infrastructure configurations remains an open challenge for practical adoption in utility asset management.

Recent advances in deep learning and UAV-based imaging have enabled substantial improvements in the automation of transmission line inspections. Convolutional neural networks and single-stage object detectors, such as You Only Look Once (YOLO), have demonstrated high accuracy in identifying electrical components and visible anomalies from aerial imagery. Building upon these developments, this work focuses on the large-scale deployment and validation of a deep learning-based inspection framework under heterogeneous real-world operating conditions.

Beyond achieving high detection accuracy, electric utility companies require inspection systems that can be reliably deployed under real operational conditions. Such systems must integrate autonomous data acquisition, robust component detection, condition assessment, and structured reporting while maintaining operational efficiency over hundreds of kilometers of transmission and distribution infrastructure. Motivated by these practical requirements, this work focuses on the development and large-scale field validation of an end-to-end inspection framework designed to support routine utility operations, emphasizing robustness, scalability, and operational applicability rather than proposing a new object detection architecture.

### Objectives and Main Contributions

The objective of this work is to develop and validate a practical framework for large-scale inspection of electrical transmission and distribution infrastructure using autonomous UAV missions and deep learning-based computer vision. Rather than proposing a new object detection architecture, this work focuses on bridging the gap between recent advances in artificial intelligence and their practical deployment in real utility inspection campaigns conducted under heterogeneous operational conditions.

The novelty of the proposed framework can be understood from three complementary perspectives: methodological, engineering, and operational.

From a methodological perspective, the proposed framework integrates multiclass object detection, dedicated condition-assessment models, and an offline asset-grouping strategy based on multi-image spatial association into a unified inspection pipeline. Unlike conventional image-level inspection approaches, the grouping stage consolidates multiple detections corresponding to the same physical asset into a unique asset-level inspection record, improving the consistency of the final inspection reports without relying on computationally intensive 3D reconstruction.

From an engineering perspective, the framework combines autonomous waypoint-based UAV missions, standardized high-resolution image acquisition, deep learning inference, and structured asset reporting into a complete end-to-end workflow suitable for large-scale inspection campaigns.

From an operational perspective, the principal contribution lies in the large-scale field validation of the proposed framework across six transmission and distribution lines located in five Brazilian states, encompassing multiple voltage levels, tower configurations, environmental conditions, and geographical regions. This extensive validation demonstrates the operational applicability and robustness of the proposed methodology under realistic inspection scenarios.

The proposed framework performs two complementary tasks: (i) the detection of electrical infrastructure components and external anomalies, and (ii) the classification of the operational condition of selected assets, including rufous-hornero nests, rust, polymeric anchoring insulators, pin-type insulators, glass disc insulators, ceramic disc insulators, and crossarms. These elements represent critical components in overhead electrical networks, as their structural integrity and proper installation directly influence system reliability and operational safety.

In addition to object detection, dedicated classification models are employed to assess the operational condition of specific components. These classification tasks include evaluating the condition of polymeric anchoring insulators (clean or dirty), identifying insulator pin attachment configurations (cable tie, elastomeric ring, untied, and plastic side tie), and assessing the structural integrity of ceramic insulator pins (normal or damaged). This multi-stage inspection strategy enables the system not only to localize infrastructure components but also to determine their condition, providing richer information to support preventive maintenance and asset management in electrical transmission and distribution systems.

Unlike previous studies that primarily focus on individual detection models, specific asset categories, or isolated inspection tasks, the proposed work delivers a complete inspection framework that integrates autonomous data acquisition, multiclass detection, condition assessment, offline asset grouping, and extensive field validation within a single operational pipeline.

By addressing the challenges of generalization, operational variability, and multiclass recognition across diverse Brazilian regions, this study consolidates deep learning-based computer vision as a practical and reliable tool for supporting utility companies in routine monitoring and preventive maintenance.

## 2. State of the Art and Related Works

The inspection of electrical transmission and distribution infrastructure has undergone substantial transformation over the last decade, driven by advances in unmanned aerial systems, remote sensing technologies, and artificial intelligence. Conventional inspection procedures based on ground crews or manned helicopters are progressively being replaced by UAVs, which offer safer operations, lower costs, and the capability to acquire high-resolution imagery from difficult-to-access viewpoints.

Recent review papers emphasize that the digitalization of power infrastructure increasingly relies on the convergence of aerial sensing technologies and artificial intelligence. In particular, Liu and Liu [9] provide a comprehensive overview of intelligent UAV-based inspection techniques for transmission lines, discussing recent advances in deep learning and sensing technologies, as well as future research directions. Similarly, Mendu and Mbuli [10] review the state of the art in UAV-based monitoring systems for power infrastructure and highlight the operational and economic benefits associated with autonomous inspections.

More broadly, the integration of aerial robotics with smart-grid technologies has received considerable attention in recent years. The survey by İnce [11] discusses how machine learning, remote sensing, and UAV technologies are transforming inspection procedures in modern electrical systems, thereby improving reliability and worker safety.

From the remote sensing perspective, photogrammetry has become one of the most widely adopted techniques for infrastructure characterization. High-resolution images acquired by UAVs enable the generation of orthomosaics, digital surface models, and three-dimensional reconstructions that provide valuable geometric information. In this context, Jiang et al. [12] proposed a high-accuracy photogrammetric framework for transmission tower reconstruction and dimensional analysis. Their methodology achieved centimeter-level accuracy, enabling precise measurements of tower geometry and conductor sag. Although reconstruction-based approaches provide excellent geometric fidelity, they require dense point-cloud generation and significant computational resources, which may limit scalability in large inspection campaigns.

Parallel to advances in remote sensing, deep learning has become the dominant paradigm for visual inspection tasks. Convolutional neural networks and object detection architectures have demonstrated remarkable performance in identifying electrical components and structural anomalies from aerial imagery. Architectures such as Faster R-CNN, SSD, and the YOLO family have been successfully applied to detect insulators, damaged hardware, corrosion, and foreign objects under challenging environmental conditions.

Recent comparative studies indicate that one-stage detectors offer an attractive balance between accuracy and computational efficiency. In particular, modern YOLO variants often achieve performance comparable to two-stage detectors while maintaining substantially lower inference times. This characteristic is especially advantageous in UAV-based inspection scenarios, where thousands of high-resolution images may be acquired during a single mission.

Recent advances in object detection have been largely driven by successive improvements within the YOLO family. Architectures such as YOLOv8, YOLOv10, and the recently introduced YOLOv11 incorporate enhanced feature extraction backbones, improved feature aggregation strategies, and optimized training procedures, resulting in increasingly favorable trade-offs between detection accuracy and computational efficiency [13,14,15]. These characteristics make modern YOLO detectors particularly attractive for large-scale UAV inspection campaigns, where thousands of high-resolution images must be processed efficiently after each mission.

In parallel, transformer-based object detectors have emerged as a competitive alternative to conventional convolutional architectures. Models such as RT-DETR eliminate the need for conventional Non-Maximum Suppression (NMS) while providing competitive inference speed through efficient transformer-based detection heads [16]. Likewise, DINO-based detectors have demonstrated remarkable localization accuracy by combining transformer attention mechanisms with denoising training strategies, establishing new state-of-the-art results on generic object detection benchmarks [17]. Although these architectures have shown excellent performance, they generally require higher computational resources and have been comparatively less explored in large-scale UAV-based inspection of electrical infrastructure.

More recently, research has also explored advanced perception strategies beyond conventional object detectors for challenging aerial and remote sensing applications. For example, Liu et al. [18] proposed an efficient detection and tracking framework for tiny airborne objects, demonstrating that the combination of lightweight feature extraction and temporal association substantially improves the robustness of UAV-based perception under challenging imaging conditions. Likewise, Fu et al. [19] introduced a unified SAM-guided self-prompt learning framework for infrared small target detection, leveraging foundation models and prompt-based representations to enhance contextual understanding and improve detection performance in low signal-to-clutter scenarios. These studies illustrate the rapid evolution of deep learning methods for aerial perception, particularly through transformer architectures, foundation models, and prompt-learning strategies. At the same time, this work focuses on integrating mature detection models into a comprehensive, field-validated inspection framework for large-scale power transmission and distribution infrastructure.

Several application-oriented studies have demonstrated the feasibility of deep learning for power infrastructure inspection. For example, Liang et al. [1] developed a convolutional neural network framework for insulator detection from UAV imagery, showing that automatic identification is feasible even under cluttered backgrounds. Likewise, Maduako et al. [2] proposed a deep-learning approach for power-line component recognition and confirmed the applicability of computer vision techniques to electrical asset inspection.

More recently, YOLO-based frameworks have become increasingly popular owing to their efficiency and ease of deployment. Chen et al. [20] investigated defect detection using UAV imagery and demonstrated that recent YOLO architectures can provide robust performance under field conditions. Similarly, Kang and Kim [21] proposed a real-time inspection framework that detects transmission-line anomalies with low computational latency.

Small-object detection remains a major challenge in aerial inspection scenarios. Components such as fittings, pins, and clamps, as well as early-stage defects, frequently occupy only a small fraction of the image. Multiscale feature extraction strategies and attention mechanisms have therefore become increasingly important for improving robustness against partial occlusions and complex backgrounds.

Besides image interpretation, autonomous navigation represents another important research direction. Recent studies have investigated sensor fusion, trajectory optimization, and safe navigation around energized structures. These approaches improve inspection efficiency while maintaining safe distances from conductors and support structures.

Despite the considerable progress achieved in autonomous navigation, photogrammetry, and deep learning-based inspection, most existing studies focus on isolated aspects of the inspection workflow. Some prioritize geometric reconstruction, whereas others focus only on single-component detection or on limited datasets. Consequently, relatively few works provide an integrated framework that combines autonomous image acquisition, multiclass component recognition, anomaly assessment, and large-scale field validation across heterogeneous environmental conditions.

Table 1 summarizes representative studies reported in the literature and positions the contribution of the present work with respect to previous approaches:

Unlike previous studies that focus on individual components or isolated stages of the inspection process, the framework proposed in this work combines autonomous UAV acquisition, multiclass component detection, condition classification, and an offline grouping mechanism that consolidates multiple observations of the same asset into a single inspection record. Furthermore, the system is validated using data acquired from six transmission and distribution lines distributed across five Brazilian states, providing a broader assessment of generalization under heterogeneous environmental and operational conditions.

## 3. Proposed System and Methodology

### 3.1. Proposed Methodology

The proposed methodology integrates autonomous UAV missions with an AI-assisted visual inspection framework that detects multiple classes of transmission and distribution assets, along with their associated anomaly states, directly from high-resolution aerial imagery.

The overall workflow comprises four main stages: (i) autonomous data acquisition, (ii) image preprocessing and standardization, (iii) multiclass object detection and component condition assessment, and (iv) post-processing and output structuring for maintenance support.

**Figure 1:**
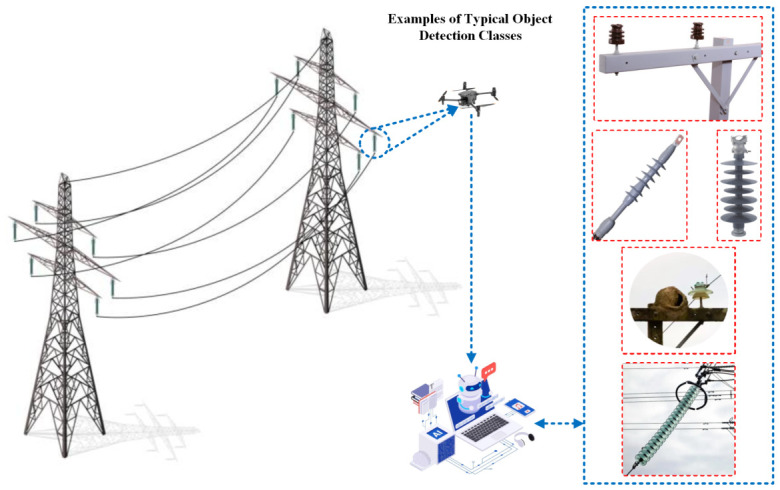
UAV performing an autonomous inspection of a high-voltage transmission line and transmitting imagery to the AI-based system for multiclass component detection.

Figure 1 presents an overview of the proposed inspection framework. The UAV autonomously acquires high-resolution imagery along the electrical corridor, and the acquired data are processed by an AI-based framework to identify infrastructure components and anomalies. This overview illustrates the end-to-end workflow described in the following subsections:

A series of field campaigns was conducted along high-voltage transmission and distribution lines, during which the UAV executed fully autonomous waypoint-based trajectories designed to maintain safe distances from towers and conductors while ensuring complete coverage of critical assets. Flight parameters, including altitude, speed, camera orientation, and lateral offset, were optimized to maximize image quality and minimize occlusions. All missions were performed in accordance with regulatory requirements and systematically documented for subsequent analysis and validation.

The effectiveness of the proposed framework strongly depends on the quality and consistency of the acquired image data. In transmission and distribution line inspections, inadequate flight strategies may negatively affect subsequent stages, including component detection, condition assessment, and anomaly identification. Therefore, the proposed methodology emphasizes mission planning, repeatability, and data standardization.

Modern UAV-based inspection systems enable autonomous mission execution by recording waypoints during an initial manual flight. Once defined, these waypoints establish a reusable inspection route that can be executed repeatedly with high positional accuracy. This capability is particularly advantageous for periodic inspections, as it ensures consistent viewpoints and enables long-term monitoring of component degradation, corrosion, structural misalignment, insulator contamination, and other anomalies.

During the initial inspection campaign, the pilot manually defines waypoints as they navigate along the Transmission Lines (TLs) and Distribution Lines (DLs), ensuring that all critical components are properly observed. The resulting waypoint sequence is then stored and reused in subsequent inspections, eliminating the need for repeated manual mission planning.

To ensure safe and reliable operation, a set of flight best practices was adopted. The most important requirement is to maintain the UAV above the energized conductors. In the proposed methodology, a vertical clearance of approximately 5 m was adopted to minimize collision risk and reduce electromagnetic interference on navigation sensors [22]. Maintaining an elevated flight profile improves stability and operational reliability.

Additionally, each maneuver, including altitude changes, lateral displacements, yaw adjustments, and turns, was explicitly represented by dedicated waypoints. This strategy avoids excessive trajectory interpolation by the mission planning, resulting in smoother, more predictable flight behavior, particularly in environments with tall metallic structures, uneven terrain, and surrounding vegetation.

Following autonomous data acquisition, the collected images were preprocessed to ensure consistency and suitability for subsequent analysis. The preprocessing stage included image selection, resolution normalization, color correction, histogram equalization under low-illumination conditions, and the removal of blurred or redundant frames. Geographical and temporal metadata were preserved to support indexing and future analysis. Unlike photogrammetric approaches, the proposed methodology does not require 3D reconstruction, thereby reducing computational complexity while preserving the visual information required for inspection tasks.

The perception module is based on a deep learning object detection architecture selected for its balance between detection accuracy and computational efficiency. In this work, the YOLOv11-M model was adopted because of its ability to perform multiclass detection in a single forward pass while maintaining compatibility with conventional GPU-based deployment environments. Its multiscale feature representation enables robust identification of components with different sizes, viewing angles, and partial occlusions, which are frequently encountered in aerial inspection scenarios.

The network was trained to recognize multiple classes of electrical infrastructure components and anomaly states. The dataset was manually annotated using bounding boxes, ensuring representative samples under different illumination conditions, backgrounds, and viewing perspectives. Furthermore, data augmentation techniques, including rotation, scaling, random cropping, and color perturbation, were employed to improve model robustness and generalization under real operating conditions.

Table 2 summarizes the composition of the dataset used in this study, including the number of annotations associated with both object detection and component condition assessment tasks. The complete dataset contains approximately 19,065 images, with each image potentially containing multiple annotated classes:

All images were manually annotated by trained specialists, with each frame individually reviewed and components of interest labeled using axis-aligned bounding boxes. To ensure annotation quality, only components occupying at least 70% of their expected visible area were considered. This threshold was empirically adopted as a conservative annotation criterion to minimize ambiguous labels while preserving representative training samples. Recent studies have shown that object detection performance deteriorates substantially under severe occlusion due to the reduced availability of discriminative visual features [23]. Targets affected by severe occlusions, motion blur, overexposure, or other image degradations were excluded from the annotation process. When multiple objects were present in the same image, all valid instances were independently annotated. Consequently, the total number of annotations exceeds the total number of images in the dataset.

The annotated dataset was divided into three subsets: training (80%), independent validation (19%), and qualitative inspection (1%). The training subset was used exclusively for model optimization. In contrast, all quantitative metrics were computed using the independent validation subset, which remained completely isolated during training. Accordingly, although referred to as the validation subset throughout this manuscript, it effectively serves as an independent test set for the final performance evaluation of the proposed framework. This partitioning strategy maximizes the amount of data available for learning while preserving an unbiased benchmark for performance evaluation.

The qualitative inspection subset was reserved exclusively for manual visual assessment. In this stage, trained annotators compared model predictions with the corresponding ground-truth labels to verify the plausibility and consistency of detections under representative inspection scenarios. Since this subset was intended only for qualitative analysis, it was not included in the metric computation.

In addition to the annotated dataset, the trained framework was deployed on a substantially larger collection of unannotated images acquired during operational field campaigns. Although no quantitative metrics could be computed for these images due to the absence of ground-truth labels, this large-scale inference stage reproduces practical deployment conditions. Further, it demonstrates the operational applicability of the proposed inspection framework.

Model training was performed using stochastic gradient descent with momentum, batch normalization, and a cosine learning rate schedule. Performance was evaluated using standard metrics, including Mean Average Precision (mAP), precision, and recall for each target class. Particular attention was given to rare anomaly classes, which naturally occur less frequently in transmission and distribution infrastructures.

After inference, the predicted outputs were refined using NMS (NMS) to remove redundant detections and consolidate confidence estimates. The resulting information was organized into a structured format containing object class, confidence score, bounding-box coordinates, and metadata extracted from the original UAV images. This structured representation facilitates the integration of inspection results into utility asset-management and preventive maintenance workflows.

### 3.2. Object Detection Architecture and Model Selection

The perception stage of the proposed framework relies on a deep learning object detector that can identify multiple classes of transmission and distribution line components and anomaly states directly from high-resolution aerial imagery. Single-stage detectors have become particularly attractive for infrastructure inspection because they provide a favorable balance between detection accuracy and computational efficiency, enabling the processing of large image datasets generated during UAV inspection campaigns.

Among the available object detection architectures, YOLO has been widely adopted in industrial inspection and remote sensing applications due to its ability to perform object localization and classification simultaneously in a single forward pass of the neural network [24,25,26]. Compared with traditional two-stage detectors, this formulation reduces computational complexity and enables efficient inference, making it particularly suitable for UAV-based inspection systems.

The perception module is based on YOLOv11-M, a single-stage object detector that offers a favorable balance between detection accuracy and computational efficiency. The network follows the conventional YOLO pipeline, consisting of three main components: a backbone for hierarchical feature extraction, a neck for multiscale feature aggregation via a feature pyramid, and a detection head that simultaneously predicts object locations, confidence scores, and class probabilities in a single forward pass. This multiscale design enables the detector to identify electrical components of different sizes while improving robustness against viewpoint changes, partial occlusions, and complex backgrounds commonly encountered in UAV-based inspections.

The medium-sized (M) variant was selected because it provides an appropriate trade-off between model complexity, inference speed, and detection accuracy for large-scale inspection campaigns involving thousands of high-resolution images. Since the proposed framework performs offline post-mission processing, this configuration offers sufficient computational efficiency while maintaining high detection performance for multiclass inspection of transmission and distribution infrastructure.

In the context of transmission and distribution line inspections, the detector is responsible for identifying multiple electrical assets and anomaly states directly from aerial imagery acquired during autonomous missions.

From a mathematical perspective, given an input image of size W×H, YOLO divides it into an S×S grid. Each grid cell is responsible for detecting objects whose centers lie within its spatial region. For each cell, the network predicts *B* bounding boxes, each represented by the vector defined in (1) [24]:

(1) $$p=(x,y,w,h,C,c_{1},c_{2},\ldots ,c_{K}),$$

where (x,y) correspond to the normalized coordinates of the bounding-box center relative to the grid cell, (w,h) represent the normalized width and height relative to the entire image, and

(2) $$C=\mathrm{Pr}(\mathrm{object})\cdot IoU_{pred,truth}$$

denotes the objectness confidence score. The terms $c_{i}$ represent the conditional class probabilities $\mathrm{Pr}({\mathrm{class}}_{i}|\mathrm{object})$ associated with each of the *K* target classes.

The overall training objective is defined by the loss function $L_{\mathrm{YOLO}}$, which combines localization, objectness, and classification losses, as expressed in (3) [24]:

(3) $$\begin{array}{cc} L_{\mathrm{YOLO}} & =\lambda_{\mathrm{coord}}\sum_{i=0}^{S^{2}}\sum_{j=0}^{B}{⊮}_{ij}^{\mathrm{obj}}\left[{\left(x_{i}-{\hat{x}}_{i}\right)}^{2}+{\left(y_{i}-{\hat{y}}_{i}\right)}^{2}+{\left(w_{i}-{\hat{w}}_{i}\right)}^{2}+{\left(h_{i}-{\hat{h}}_{i}\right)}^{2}\right]\\ & +\sum_{i=0}^{S^{2}}\sum_{j=0}^{B}{⊮}_{ij}^{\mathrm{obj}}{\left(C_{i}-{\hat{C}}_{i}\right)}^{2}+\lambda_{\mathrm{noobj}}\sum_{i=0}^{S^{2}}\sum_{j=0}^{B}{⊮}_{ij}^{\mathrm{noobj}}{\left(C_{i}-{\hat{C}}_{i}\right)}^{2}\\ \; & +\sum_{i=0}^{S^{2}}{⊮}_{i}^{\mathrm{obj}}\sum_{k=1}^{K}{\left(p_{i}\left(k\right)-{\hat{p}}_{i}\left(k\right)\right)}^{2}, \end{array}$$

where $⊮*{ij}^{\mathrm{obj}}$ indicates whether the *j*-th bounding-box predictor in cell *i* is responsible for detecting an object. The hyperparameters λ∗coord and $\lambda_{\mathrm{noobj}}$ control the relative importance of localization errors and background confidence penalties during training.

In this work, the YOLOv11-M variant implemented in the Ultralytics framework was adopted due to its favorable trade-off between model capacity and computational efficiency. The medium-scale architecture and multiscale feature aggregation enable robust detection of components with different sizes, viewpoints, and partial occlusions, which are common characteristics of aerial inspection imagery.

The Intersection over Union (IoU) is a fundamental metric used to quantify the overlap between predicted bounding boxes and ground-truth annotations [24,27]. It is defined as

(4) $$IoU=\frac{\left|A\cap B\right|}{\left|A\cup B\right|},$$

where $\left|A\cap B\right|$ represents the intersection area between the predicted and reference bounding boxes, and $\left|A\cup B\right|$ corresponds to their union.

Model performance was evaluated using precision and recall. Precision measures the proportion of correctly predicted objects among all detections, whereas recall quantifies the proportion of ground-truth objects successfully identified by the model [28]:

(5) $$P=\frac{TP}{TP+FP},$$

(6) $$R=\frac{TP}{TP+FN},$$

where TP denotes true positives, FP false positives, and FN false negatives.

To provide a comprehensive assessment across all classes, the mAP metric was adopted. The mAP corresponds to the average of the Average Precisions (APs) computed for each class:

(7) $$\mathrm{mAP}=\frac{1}{N}\sum_{i=1}^{N}{\mathrm{AP}}_{i},$$

where *N* is the total number of classes and ${\mathrm{AP}}_{i}$ denotes the average precision associated with the *i*-th class.

In this study, the ${\mathrm{mAP}}_{50}$ metric was adopted using an IoU threshold of 0.5 between predicted and ground-truth bounding boxes [29]. This criterion determines whether a detection is considered correct based on the spatial overlap between the predicted object and its corresponding annotation.

### 3.3. Summary of Methodology

Overall, the proposed framework provides a lightweight and scalable solution for transmission and distribution line inspection by combining autonomous UAV-based image acquisition with AI-assisted multiclass component detection and condition assessment. The methodology was designed to operate under real inspection conditions while avoiding the computational overhead associated with 3D reconstruction approaches.

The dataset’s diversity, collected across multiple geographical regions and operating environments, contributes to improved robustness and generalization under heterogeneous field conditions. Furthermore, integrating autonomous mission execution, standardized image acquisition, object detection, and condition assessment yields an end-to-end inspection workflow suitable for preventive maintenance and asset management applications.

Table 3 summarizes the main hyperparameters adopted during the training process:

## 4. Large-Scale Field Deployment

This section describes the operational scenario in which the proposed inspection framework was deployed and evaluated. The framework combines autonomous UAV missions with an AI-based visual analysis pipeline designed for large-scale inspection of transmission and distribution infrastructures.

**Figure 2:**
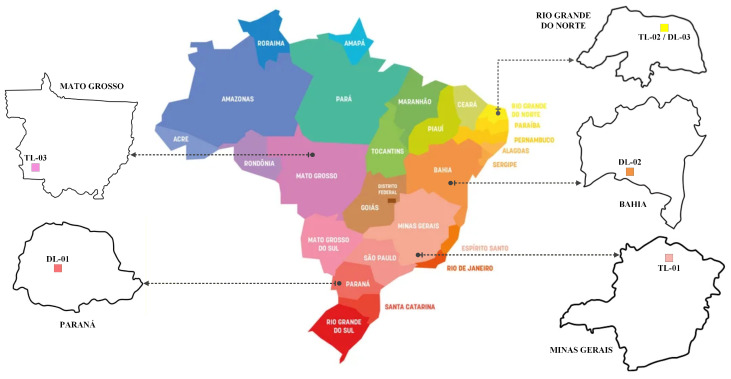
Geographical distribution of the TLs and DLs considered in this study. The figure highlights the Brazilian states where inspections were conducted, and the locations of each analyzed line segment.

The field campaigns were conducted across six transmission and distribution lines located in different regions of Brazil, as illustrated in Figure 2:

The selected lines encompass different voltage levels, structural configurations, terrain characteristics, and environmental conditions, reflecting the variability typically encountered in real-world power systems.

This diversity is particularly important for evaluating the robustness of the proposed framework under heterogeneous operating conditions. By covering multiple electrical corridors and geographical regions, the inspection campaigns provide evidence of the framework’s applicability beyond a single location or infrastructure configuration.

Table 4 summarizes the main characteristics of the transmission and distribution lines considered in this study, including voltage levels, line lengths, number of conductors, and the total number of support structures inspected. The Brazilian states are identified using their standard abbreviations:

In total, the field campaigns covered approximately 231.2 km of electrical infrastructure and 2925 support structures distributed across five Brazilian states. This large-scale deployment enabled data collection across diverse operational scenarios and provided a representative basis for evaluating the proposed inspection framework.

To further characterize the heterogeneity of the inspection environments, Table 5 complements Table 4 by presenting additional descriptors associated with each line, including tower density, biome or terrain type, and the predominant visual challenges encountered during image acquisition:

The inspection scenarios include mountainous regions, coastal areas, tropical environments, and semi-arid landscapes. Consequently, the acquired dataset incorporates a wide range of background conditions, vegetation profiles, illumination levels, atmospheric effects, and structural configurations. These characteristics enable assessment of the framework under realistic field conditions and support its ability to generalize across distinct inspection scenarios.

## 5. Artificial Intelligence Framework

This section describes the artificial intelligence framework adopted for the automated inspection of electrical assets associated with transmission and distribution lines. Rather than proposing a new neural network architecture, this work aims to operationalize and validate a complete inspection framework capable of supporting large-scale field deployments under heterogeneous real-world conditions. Consequently, the emphasis of this work lies on robustness, scalability, and practical deployment rather than on architectural modifications to existing deep learning models.

Before presenting the learning architecture, it is important to establish the functional requirements that guided the design of the proposed framework.

The inspection framework was designed to perform large-scale visual analysis of electrical assets distributed along transmission and distribution corridors. Since inspection campaigns involve thousands of structures and large image volumes acquired under variable field conditions, the system must be sufficiently robust and adaptable to operate reliably across distinct geographical regions and infrastructure configurations.

Unlike approaches that rely on a single reference structure or require computationally intensive 3D reconstruction procedures, the proposed framework performs component identification directly from high-resolution aerial imagery. This design choice reduces computational complexity and facilitates large-scale deployment while preserving the visual information required for inspection tasks.

The framework can detect multiple classes of electrical assets and their associated anomaly states. The inspected elements include support structures, insulators, hardware fittings, crossarms, and other auxiliary components distributed throughout towers, poles, and conductors. Since these elements are spatially distributed across the electrical infrastructure, the inspection process is distributed, without relying on predefined reference objects.

In addition to object localization, the framework includes dedicated condition assessment stages to identify visual indicators of abnormal operating conditions. These include material degradation, corrosion, contamination, structural damage, and installation-related anomalies that may affect system reliability and operational safety.

For each identified anomaly, the framework generates structured visual evidence associated with the corresponding asset. This information supports preventive and condition-based maintenance strategies by enabling remote inspection and reducing the dependence on manual procedures in hazardous or difficult-to-access environments.

Therefore, the proposed framework integrates autonomous data acquisition, multiclass object detection, and component condition assessment into an inspection workflow suitable for utility asset management applications and large-scale field deployments.

### 5.1. Object Detection Framework

The core perception module of the proposed inspection framework is based on the YOLO family of object detectors, which has been widely adopted in power line inspection applications due to its favorable balance between accuracy and computational efficiency [1,2].

Unlike two-stage approaches, YOLO formulates object detection as a single-stage regression problem, simultaneously predicting object locations and class probabilities in a single forward pass [24]. This characteristic makes the architecture particularly suitable for UAV-based inspection scenarios, where large volumes of high-resolution images must be processed efficiently.

The primary objective of this work is not to propose a new object detector or perform a systematic comparison among deep learning architectures. Instead, the focus is on developing and validating an end-to-end inspection framework that can operate under heterogeneous real-world conditions.

Accordingly, the YOLO family was selected because of its maturity, extensive adoption in related transmission line inspection studies, and demonstrated robustness in multiclass detection tasks [1,2]. The use of an established architecture also facilitates comparisons with previous studies. It emphasizes that the contribution of this work lies in the inspection methodology and its operational deployment rather than in architectural modifications.

Among the available variants, YOLOv11-M was adopted during the development phase of this research due to its most recent, stable implementation and its favorable compromise between model capacity and computational cost. The medium-scale architecture provides sufficient representational power to detect components of varying sizes and viewpoints while maintaining practical inference times on large image datasets.

The detector was configured to support multiclass recognition of electrical assets and anomaly states under varying illumination conditions, complex backgrounds, partial occlusions, and viewpoint variations typically encountered during field inspections.

A dedicated dataset was constructed using images acquired during autonomous UAV missions performed over multiple transmission and distribution lines. The dataset encompasses diverse environmental conditions, tower geometries, and asset configurations, providing representative samples of real inspection scenarios. All images were manually annotated using bounding boxes and class labels to ensure high-quality supervision during training.

### 5.2. Object Grouping and Multi-Image Association

During transmission line inspections, the same electrical component frequently appears in multiple images acquired from different viewpoints. If each image were analyzed independently, the same asset could be counted multiple times, resulting in redundant inspection records and unnecessary maintenance reports.

To address this issue, an object grouping stage was incorporated into the proposed framework. Rather than treating each detection as an independent event, the system associates detections corresponding to the same physical asset and consolidates them into a single inspection record.

The grouping process is executed after the YOLO inference stage and receives both the detected bounding boxes and the metadata associated with each image. These metadata include the camera position, orientation, and acquisition parameters obtained during the autonomous UAV mission. Such information enables projecting image-level detections into a common spatial reference, allowing multiple observations of the same component to be associated independently of their positions within the image sequence.

The grouping procedure relies on three complementary criteria:

- Identical object class;
- Spatial consistency between projected detections;
- Availability of image metadata describing the camera pose and acquisition geometry.

When these conditions are satisfied, detections are merged into a single group representing one physical component. For each group, the system stores only one final record while preserving the highest-confidence detection and the metadata associated with all supporting images.

Unlike online object-tracking approaches based on Kalman filtering, optical flow, or multi-object trackers, the proposed association mechanism does not rely on temporal continuity across consecutive frames. Instead, grouping is performed offline after image acquisition by exploiting the spatial relationships derived from the image metadata. This strategy considerably simplifies computational requirements while providing a robust asset-level representation suitable for large-scale transmission line inspections.

**Figure 3:**
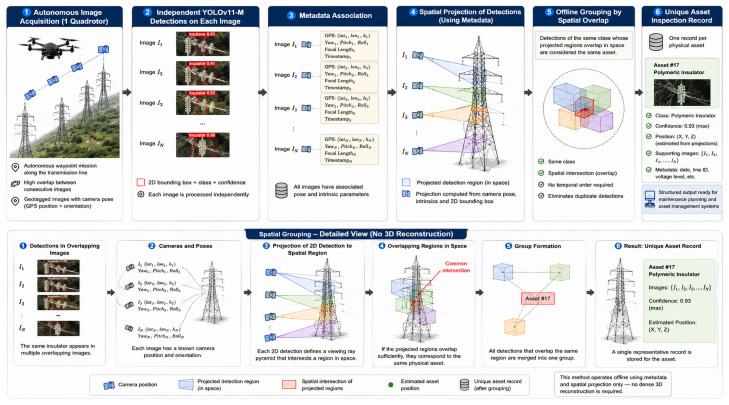
Overview of the proposed offline asset grouping procedure. Independent detections from overlapping UAV images are projected into a common 3D spatial reference and associated according to class consistency and spatial proximity. Multiple observations of the same physical component are merged into a single inspection record, reducing redundant detections and improving database consistency.

Figure 3 illustrates the overall grouping workflow adopted in this work:

As illustrated in Figure 3, the grouping stage acts as an intermediate layer between image-level inference and asset-level reporting. Instead of treating detections independently, the proposed method consolidates multiple observations obtained from overlapping UAV images into a single representation of the corresponding physical asset. This strategy reduces redundancy and improves the consistency of inspection reports, particularly in transmission line environments where the same component may be observed from different viewpoints during autonomous flights.

Independent detections are first projected into a common spatial reference using the image metadata associated with each frame. Subsequently, detections belonging to the same class and exhibiting spatial consistency are associated and merged into a single asset instance. The final representation preserves the highest-confidence detection together with references to all supporting images contributing to that asset.

This asset-oriented representation offers several practical advantages. First, it prevents multiple counting of the same component during large inspection campaigns involving thousands of images. Second, it simplifies the information delivered to maintenance teams by generating concise and structured inspection reports. Finally, it improves database consistency by establishing a one-to-one correspondence between physical assets and digital inspection records, facilitating integration with maintenance databases and asset management systems.

It is important to emphasize that the proposed grouping strategy is intended for asset association rather than precise geometric reconstruction. Unlike photogrammetric approaches based on dense 3D models, the framework uses the image metadata only to project detections into a common spatial reference, enabling the association of multiple observations of the same physical asset. Consequently, moderate positioning inaccuracies resulting from UAV navigation or camera pose estimation do not compromise the objective of generating a unique inspection record for each asset. Applications requiring centimeter-level geometric measurements, such as conductor sag estimation or clearance analysis, remain better suited to dedicated 3D reconstruction workflows.

### 5.3. Evaluation Metrics

The performance of the proposed framework was evaluated on the independent validation dataset using Precision (P), Recall (R), and mean Average Precision at an Intersection over Union threshold of 50% (mAP_50_). The mathematical definitions of these metrics were introduced in Section 3.2.

The mAP_50_ metric was adopted as the primary performance indicator because it provides a balanced measure of detection accuracy across all classes and is widely used in object detection benchmarks.

Together, these metrics provide complementary information regarding detection accuracy and robustness across different component categories and inspection scenarios. The corresponding quantitative results are presented in Section 6.

### 5.4. Training and Inference Configuration

The design choices adopted in the proposed framework were guided by established practices for object detection in real-world and class-imbalanced datasets.

The annotated dataset was partitioned at the structure level, ensuring that all images associated with the same transmission tower and its corresponding infrastructure components were assigned exclusively to either the training or the validation subset. Consequently, images depicting the same physical asset from different viewpoints were never shared between the two subsets, preventing information leakage and enabling a more rigorous assessment of the model’s generalization capability under real inspection conditions.

The primary objective of this study is to conduct large-scale field validation of the complete inspection framework under operational conditions, rather than to optimize its individual modules. Consequently, a formal ablation study was not conducted. Since the proposed pipeline combines independently trained detection and condition assessment models with an offline asset grouping stage operating at the reporting level, a comprehensive ablation analysis would require dedicated asset-level evaluation protocols beyond the image-level detection metrics considered in this work.

Detections are performed independently for each image, without using temporal tracking algorithms. This design simplifies the processing pipeline and facilitates large-scale deployment while preserving computational efficiency.

The offline asset grouping stage subsequently associates detections corresponding to the same physical component using spatial projections of detections derived from image metadata, thereby eliminating redundant inspection records without affecting image-level detection performance.

The training dataset exhibits a naturally imbalanced class distribution, reflecting the frequency with which different electrical components and anomalies occur during real inspection campaigns. Rather than artificially balancing the dataset through over- or undersampling, the original class distribution was preserved to maintain consistency with operational scenarios. Data augmentation strategies, including rotation, scaling, color jittering, and random cropping, were applied to increase intra-class variability and improve robustness against viewpoint and illumination changes. In addition, background-only images were incorporated as negative samples to reduce false positives, with their proportion limited to approximately 10% of the training set to avoid degrading the performance of underrepresented classes. Despite the class imbalance, the results presented in Section 6 indicate that the proposed framework achieved consistently high detection performance across all classes, while the comparatively lower accuracy observed for visually variable classes, such as rust and rufous-hornero nests, is attributed primarily to their greater appearance variability rather than solely to the number of training samples.

All performance metrics reported in this work were computed using a confidence threshold of 0.25 and an NMS IoU threshold of 0.70. These values were selected after preliminary experiments on the validation set to provide a suitable balance between precision and recall while preserving detections in densely populated structures, such as insulator strings and closely spaced components. Although a comprehensive threshold sensitivity analysis could provide additional insights, it falls slightly beyond the scope of the present work, whose primary objective is the large-scale field validation of the proposed inspection framework.

## 6. Experimental Results and Discussion

The experimental evaluation was designed to assess both detection accuracy and operational robustness under heterogeneous real-world conditions. The validation dataset comprises images collected from multiple voltage levels, tower configurations, climatic conditions, and environmental backgrounds, providing a representative assessment of the framework under realistic inspection scenarios.

This section presents the quantitative and qualitative evaluation of the proposed inspection framework. The experiments were conducted using a multi-regional dataset acquired across six transmission and distribution lines (Table 4), enabling the assessment of the framework under diverse geographical, structural, and environmental conditions.

The evaluation emphasizes not only object detection accuracy but also the framework’s ability to maintain consistent performance across heterogeneous operating scenarios. This characteristic is particularly important for large-scale inspection applications, where image characteristics and infrastructure configurations vary significantly between regions.

### 6.1. Quantitative Performance Analysis

The performance of the detection and classification stages was evaluated using standard metrics, including mean Average Precision (mAP_50_), Precision, Recall, and the distribution of True Positives (TP), False Positives (FPs), and False Negatives (FNs).

#### 6.1.1. Object Detection Performance

**Figure 4:**
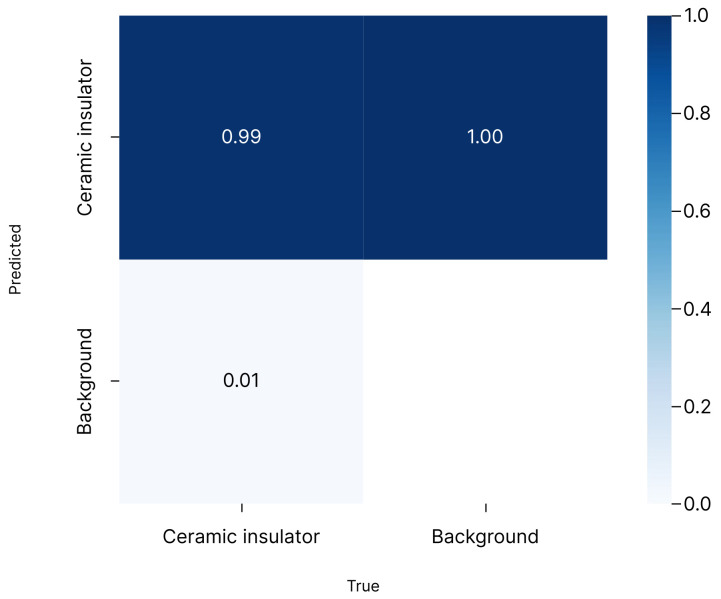
Ceramic insulator confusion matrix normalized.

**Figure 5:**
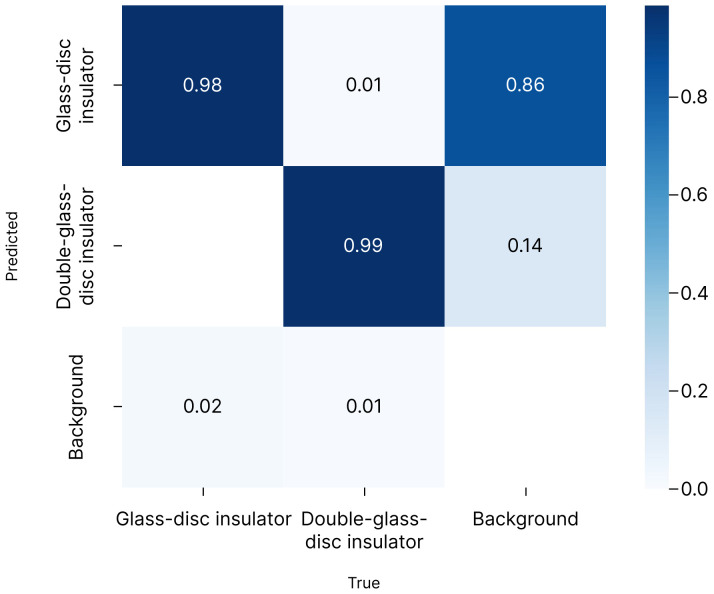
Glass disc insulator confusion matrix normalized.

**Figure 6:**
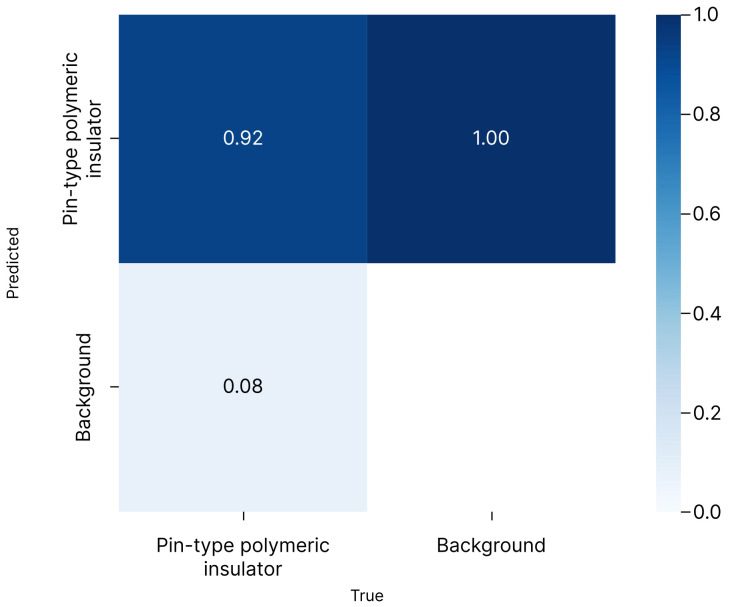
Pin-type polymeric insulator confusion matrix normalized.

**Figure 7:**
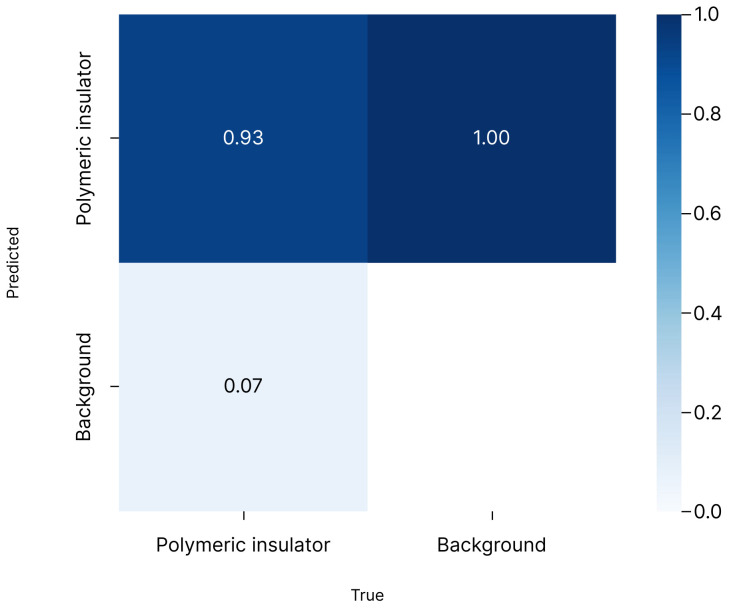
Polymeric insulator confusion matrix normalized.

**Figure 8:**
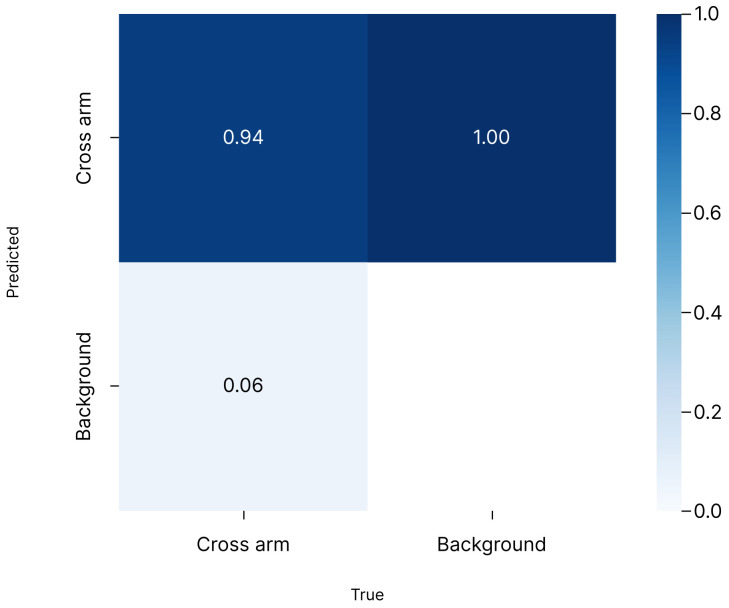
Crossarm confusion matrix normalized.

**Figure 9:**
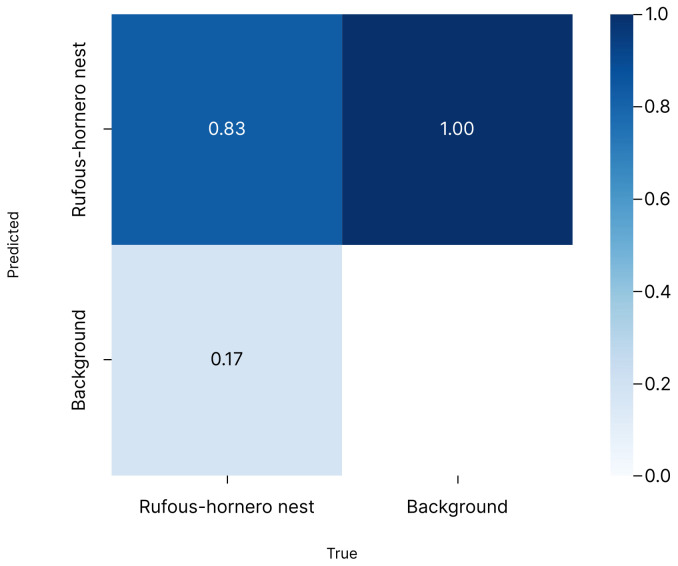
Rufous-hornero nest confusion matrix normalized.

**Figure 10:**
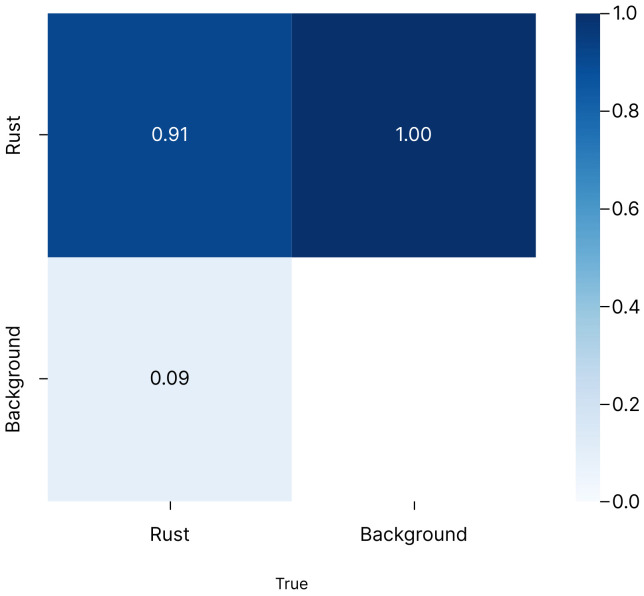
Rust confusion matrix normalized.

Table 6 summarizes the quantitative results obtained for the multiclass component and anomaly detection. The reported metrics were computed using the independent validation subset described in Section 5.4:

The proposed framework demonstrated strong performance in identifying insulating assets. Ceramic disc insulators and glass disc insulators achieved mAP_50_ scores of 0.9935 and 0.9817, respectively, with recall values of 0.9802 and 0.9648. The corresponding confusion matrices (Figure 4 and Figure 5) indicate high true-positive rates with limited background confusion, suggesting that the detector successfully captures the characteristic geometric and textural features of these components, even under complex background conditions:

The pin-type insulator also exhibited strong detection performance, achieving an mAP_50_ of 0.9788 and a precision of 0.9810 (Figure 6). Similarly, the polymeric anchoring insulator reached an mAP_50_ of 0.9645 while handling the largest validation subset among all classes (2399 instances), as illustrated in Figure 7:

These results indicate that the framework maintains stable performance across both common and high-frequency asset categories. Then, the crossarm class achieved an mAP_50_ of 0.9627 (Figure 8), demonstrating the capability of the framework to detect structural support elements under different tower geometries and inspection scenarios:

Regarding anomaly detection, the framework achieved an mAP_50_ of 0.9245 for rufous-hornero nests (Figure 9) and 0.8945 for rust detection (Figure 10):

Rust detection proved to be one of the most challenging tasks because corrosion patterns exhibit considerable visual variability and may resemble soil coloration, shadows, mud deposits, dust, or natural surface texture variations. Nevertheless, the framework achieved a precision of 0.8990, indicating its ability to identify appearance-variable anomalies under real field conditions.

Although corrosion is an important indicator of asset condition, the operating point adopted in this work was intentionally selected to balance recall and precision rather than maximizing sensitivity alone. In practical UAV-based inspections, increasing detector sensitivity to reduce FNs inevitably results in a substantial increase in FP detections, as environmental artifacts such as dust, soil, mud, and naturally occurring reddish or brown stains often exhibit visual characteristics similar to those of corrosion. During discussions with the participating utility, minimizing unnecessary maintenance inspections was identified as a key operational requirement, since excessive false alarms increase inspection workload and reduce operator confidence in AI-assisted inspection systems. Therefore, the reported performance reflects a practical trade-off for large-scale field deployment. At the same time, periodic inspection campaigns offer additional opportunities to detect ambiguous or early-stage corrosion before it evolves into a critical structural issue.

#### 6.1.2. Condition Classification Performance

**Figure 11:**
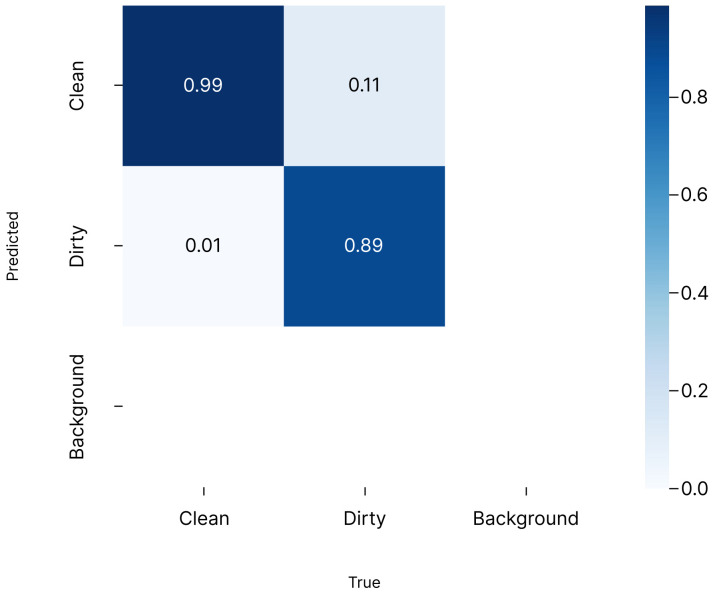
Polymeric insulator classification confusion matrix normalized.

**Figure 12:**
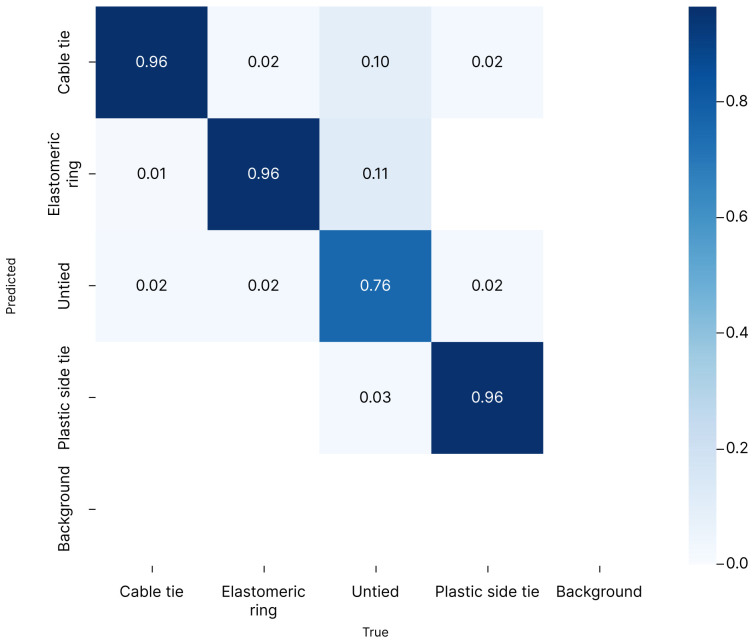
Pin-type classification confusion matrix normalized.

The performance of the classification models used for condition assessment is summarized in Table 7:

The proposed framework achieved an overall accuracy of 0.97 in distinguishing between clean and dirty polymeric anchoring insulators. The corresponding confusion matrix (Figure 11) indicates limited misclassification between both conditions:

The classification of insulator pin attachment configurations (cable tie, elastomeric ring, untied, and plastic side tie) achieved an overall accuracy of 0.92, as shown in Figure 12:

The results above indicate that the classifier can distinguish subtle structural differences among attachment types, including configurations associated with improper installation or missing fastening elements.

**Figure 13:**
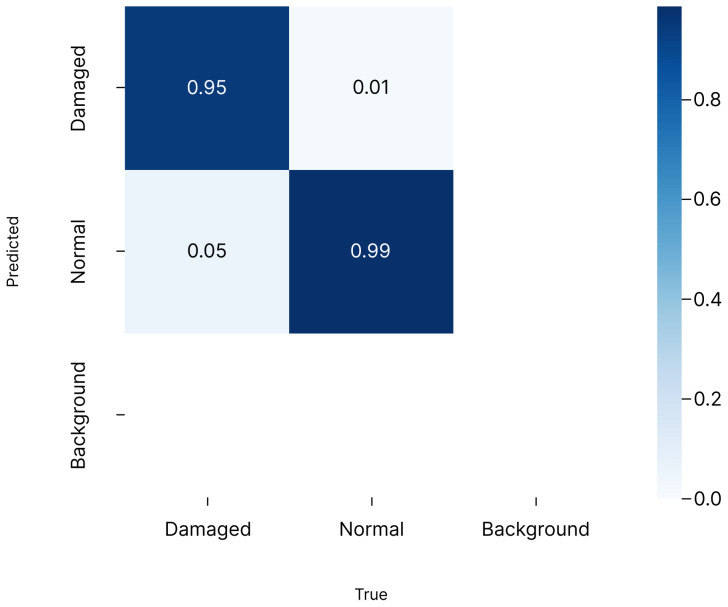
Ceramic insulator classification confusion matrix normalized.

For ceramic insulator pins, the classification model achieved an overall accuracy of 0.98. Figure 13 shows the corresponding confusion matrix, indicating a low number of misclassifications between normal and damaged components. Accurate identification of damaged pins is particularly relevant because these defects may compromise the mechanical integrity of the insulator assembly and increase the risk of service interruptions:

Overall, the classification stage complements the object detection module by providing condition information for selected assets, enabling a more detailed assessment of component integrity and supporting preventive maintenance strategies in transmission and distribution systems.

### 6.2. Qualitative Analysis and Error Discussion

A qualitative review of the model outputs provides additional insight into the behavior of the proposed framework under real inspection conditions, particularly regarding representative successful detections and the occurrence of FPs and FNs.

#### 6.2.1. Successful Inferences and Generalization

**Figure 14:**
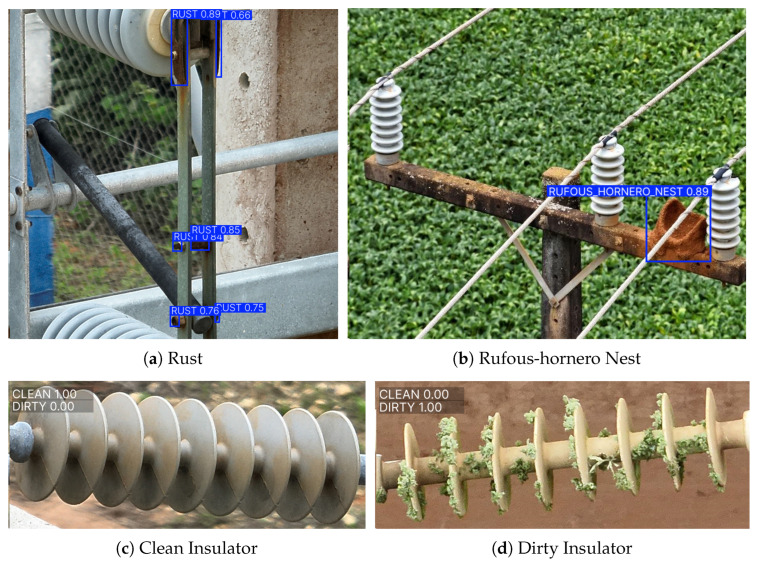
Examples of successful inferences produced by the proposed inspection framework.

The framework maintained consistent detection performance across distinct geographical regions, including the semi-arid environments of Bahia (BA) and the mountainous areas of Minas Gerais (MG). Representative successful detections are characterized by accurate object localization and confidence scores compatible with the visual quality of the inspected assets under different illumination conditions and viewing angles, as illustrated in Figure 14:

The large number of correctly detected instances for ceramic disc insulators, glass disc insulators, and polymeric anchoring insulators indicates that the framework maintains stable performance across classes, even with a high number of samples and diverse acquisition conditions.

#### 6.2.2. Analysis of False Positives (FP)

**Figure 15:**
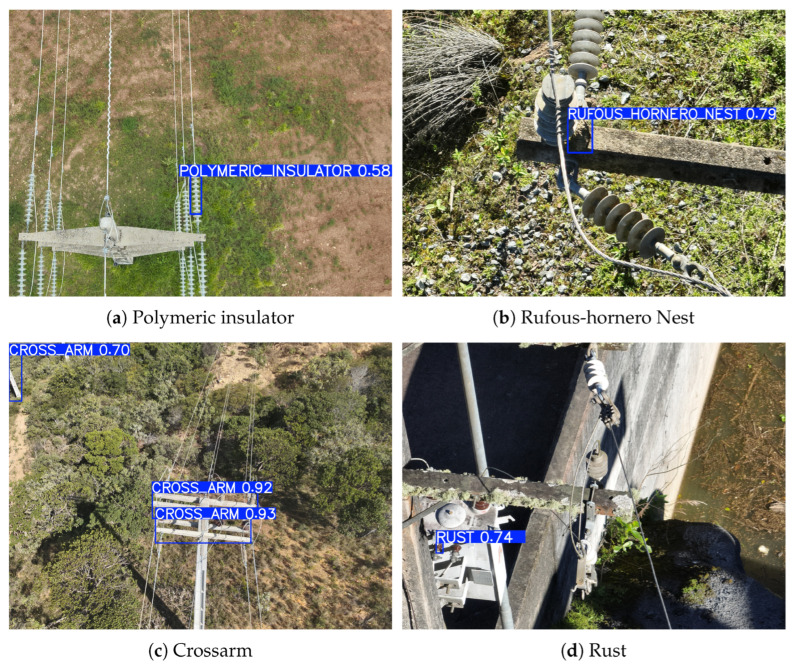
Examples of false-positive detections produced by the proposed inspection framework.

Among the detection classes, the largest number of false positives was observed for polymeric anchoring insulators (FP = 162) and glass disc insulators (FP = 228). In the case of polymeric insulators, most false detections occurred when elongated metallic components or structural hardware presented visual characteristics similar to those of the target class. For glass disc insulators, false positives were mainly due to visual ambiguity between single- and double-disc configurations at specific viewpoints. Representative examples are presented in Figure 15:

For the rust class, 74 false positives were observed. Most of these cases involve reddish-brown surface deposits, soil residues, or natural discoloration patterns that exhibit visual characteristics similar to those of corrosion. Such behavior reflects the intrinsic difficulty of distinguishing early-stage corrosion from environmental surface artifacts, particularly under uneven illumination and varying background textures.

#### 6.2.3. Analysis of False Negatives (FNs)

**Figure 16:**
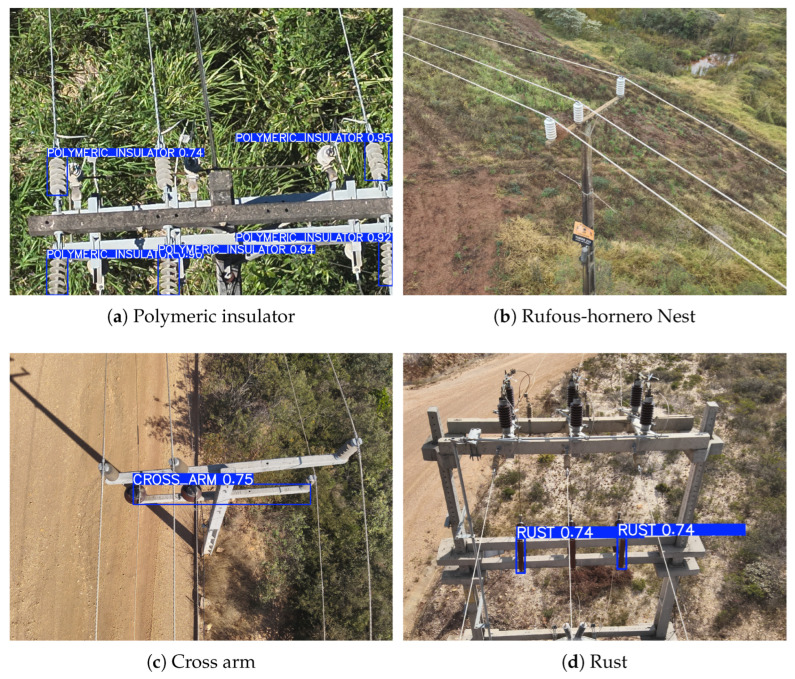
Examples of false-negative detections produced by the proposed inspection framework.

Among the detection classes, the largest number of false negatives was observed for polymeric anchoring insulators (FN = 163), followed by rufous-hornero nests (FN = 20), as illustrated in Figure 16:

For polymeric anchoring insulators, most false negatives are associated with severe occlusions caused by tower elements, conductors, or unfavorable viewpoints. Additional missed detections were observed under low-contrast conditions, where the insulator’s visual appearance partially blends with the sky background.

For rufous-hornero nests, the irregular morphology of the structures and their relatively small apparent size in wide-angle UAV images may lead to missed detections. These effects become more pronounced under direct sunlight or when conductors and surrounding structures partially occlude the nests.

In the pin attachment classification task, the untied configuration exhibited the largest number of false negatives (FN = 19). Because the distinction between tied and untied configurations may correspond to only a few pixels at typical UAV operating distances, image quality, illumination conditions, and oblique viewing angles can make it more difficult to classify these cases correctly.

Overall, the observed false negatives are primarily associated with partial occlusions, small target dimensions, and challenging imaging conditions, which are common limitations in aerial inspection scenarios. Despite these challenges, the framework maintained stable performance across the evaluated classes and operating conditions.

### 6.3. Operational Impact and Scalability

The results obtained in this study indicate that the proposed framework can support large-scale inspection of electrical infrastructure while maintaining high detection accuracy and moderate computational requirements.

The proposed pipeline performs offline post-mission inference. After image acquisition, the collected data are transferred to a ground workstation for processing, thereby eliminating the need for strict onboard computational requirements. The experiments were performed on a workstation equipped with an NVIDIA RTX 4090 GPU. The YOLOv11-M model achieved an average inference latency of 15.5 ms per image, corresponding to approximately 64.5 FPS and a theoretical throughput of about 3870 images per minute during the detection stage. GPU memory consumption during inference (batch size = 1) was approximately 1.5–2.0 GB of VRAM, enabling efficient processing of large inspection datasets on commercially available high-performance hardware. Since inference is performed offline, the overall processing time scales according to the available computational resources and the required inspection throughput.

The structured outputs generated by the framework, which include object classes, confidence scores, localization information, and associated metadata, support the transition from conventional periodic inspections to condition-based maintenance strategies. By automatically identifying components that require attention, the system enables utility operators to prioritize interventions based on objective visual evidence.

Furthermore, the proposed approach improves operational safety by reducing field personnel’s exposure to energized structures and hard-to-access locations. Since image acquisition is performed remotely through autonomous UAV missions, many inspection activities that traditionally require direct human intervention can be replaced by digital analysis workflows.

The multi-regional composition of the dataset reflects a deliberate design choice to evaluate the framework under a broad spectrum of structural and environmental conditions. As detailed in Table 4 and Table 5, the six inspected lines span voltage levels ranging from 34.5 kV to 500 kV, tower densities from 2.3 to 44.6 towers/km, and distinct Brazilian environmental conditions. No region-specific fine-tuning or domain adaptation was applied. Instead, the same YOLOv11-M model, trained once using the fixed hyperparameters presented in Table 3, was deployed uniformly across all line segments. The resulting mAP_50_ of 0.9572 across seven detection classes provides empirical evidence of the framework’s ability to generalize across heterogeneous operational scenarios.

To quantify the practical benefits of the proposed framework, a cost and productivity analysis was conducted using operational data from Elera Renewable Energies. The analysis compares the proposed UAV-based methodology with two conventional inspection approaches currently employed by the company: outsourced inspections performed by specialized contractors and traditional manual inspections executed by internal maintenance teams.

For transmission line inspections, outsourced services cost approximately US$392.60 per kilometer on average. Internal manual inspections require approximately 5.0 human-hours per kilometer, resulting in an estimated cost of US$81.50 per kilometer. In contrast, the proposed UAV-based methodology requires approximately 0.6 human-hours per kilometer, corresponding to an estimated labor cost of US$9.80 per kilometer.

Table 8 summarizes the comparison among the evaluated inspection strategies:

Based on these values, the proposed methodology reduced inspection costs by approximately 97.5% compared with outsourced services and by 88.0% compared with conventional internal inspections for the industrial case study considered in this work. Likewise, the required labor effort decreased from 5.0 to 0.6 human-hours per kilometer, corresponding to an approximately 8.3-fold increase in operational productivity. These results are based on operational data from Elera Renováveis and therefore reflect the inspection workflow adopted by that utility.

Since detailed operational costs and productivity indicators are typically considered proprietary information and are rarely disclosed publicly by utility companies, comprehensive economic comparisons across different operators are seldom available in the literature. Although the proposed framework is not free from detection errors, the operational gains observed during large-scale deployment demonstrate that the methodology already provides substantial practical value for supporting routine inspection activities when compared with the conventional procedures currently employed by the participating utility.

Additional contractual data from generation assets indicate cumulative inspection expenditures exceeding US$248,000 annually, corresponding to approximately US$995,000 over a four-year maintenance cycle. These values illustrate the potential economic impact of incorporating automated inspection technologies into routine maintenance activities.

Beyond direct cost reductions, the framework offers additional operational advantages, including increased inspection frequency, standardized digital records, georeferenced documentation, faster anomaly identification, and reduced personnel exposure to hazardous environments.

Overall, the results suggest that the proposed framework can help reduce inspection costs, increase productivity, and improve personnel safety while preserving the quality and repeatability of inspection records. These characteristics support its adoption in large-scale transmission, distribution, and generation asset management applications.

## 7. Conclusions

This work presented a large-scale, field-validated framework for automated inspection of high-voltage transmission and distribution line infrastructure, combining autonomous UAV missions with an AI-based multiclass detection and classification pipeline. The principal contribution of this work lies in the large-scale validation of an integrated UAV and deep learning inspection framework, rather than in introducing a new neural network architecture. The primary objective was to develop, validate, and deploy a robust computer vision system capable of identifying critical electrical components and structural anomalies across diverse geographic and environmental contexts in Brazil, without relying on computationally intensive 3D reconstruction workflows.

The proposed system was deployed and validated across six transmission and distribution lines spanning five Brazilian states (Minas Gerais (MG), Rio Grande do Norte (RN), Mato Grosso (MT), Paraná (PR), and Bahia (BA)), covering a total of 2925 support structures and line segments ranging from 10.7 km to 78.9 km, at voltage levels between 34.5 kV and 500 kV. This multi-regional coverage allowed the framework to be evaluated under a broad spectrum of structural configurations, environmental conditions, and imaging scenarios, providing a rigorous assessment of its generalization capability.

The resulting dataset comprised approximately 19,065 annotated images and more than 36,000 object annotations distributed across seven detection classes and eight condition-classification categories. Overall, the proposed detection framework achieved a mean mAP_50_ of 0.9572 across seven detection classes. In contrast, the dedicated classification models achieved accuracies ranging from 0.92 to 0.98 depending on the evaluated asset condition.

The detection model, based on the YOLOv11-M architecture, demonstrated strong performance across all target classes. Among insulating components, the ceramic disc insulator achieved the highest detection accuracy, with mAP_50_ of 0.9935, precision of 0.9813, and recall of 0.9802. The glass disc insulator and the pin-type insulator also yielded high mAP_50_ values of 0.9817 and 0.9788, respectively. The polymeric anchoring insulator, evaluated over the largest validation set (2399 instances), reached mAP_50_ of 0.9645, confirming the framework’s scalability. For structural components, the crossarm class achieved mAP_50_ of 0.9627. In anomaly detection, rufous-hornero nests were identified with mAP_50_ of 0.9245, while rust, the most visually variable class, reached mAP_50_ of 0.8945 with a precision of 0.8990.

Beyond component detection, the dedicated classification models further enhanced the framework’s diagnostic capability. The polymeric insulator condition classifier (clean versus dirty) achieved an overall accuracy of 0.97. The pin attachment configuration classifier, which distinguishes four installation states, reached an accuracy of 0.92. The ceramic insulator pin integrity model achieved the highest classification accuracy (0.98), demonstrating its potential for supporting early identification of structural failures.

Unlike many previous studies that focus primarily on algorithmic modifications or controlled datasets, the present work emphasizes operational deployment and large-scale validation. The proposed framework combines autonomous data acquisition, multiclass detection, condition assessment, and an offline grouping strategy that consolidates repeated detections into unique asset records. Together, these modules form a unified inspection workflow evaluated under heterogeneous real-world conditions.

Collectively, the results indicate that the proposed framework can reliably identify and assess the condition of electrical infrastructure assets across diverse operating scenarios. By eliminating the need for 3D reconstruction, enabling autonomous image acquisition, and producing structured outputs compatible with asset management systems, the framework provides a practical pathway toward condition-based maintenance in the electrical transmission and distribution sector.

The system’s ability to reduce field crew exposure while maintaining high detection accuracy further reinforces its value for improving operational safety and infrastructure reliability. The economic assessment further indicated that the proposed methodology can reduce inspection costs by up to 97.5% relative to outsourced inspection services and by approximately 88.0% compared with conventional in-house inspections. In addition, the required labor effort decreased from 5.0 to 0.6 human-hours per kilometer, corresponding to an approximately 8.3-fold increase in operational productivity. These results further support the practical feasibility of large-scale deployment.

### Future Works

Several directions are identified for extending the proposed framework. The detection dataset could be expanded to include additional component classes and anomaly types not covered in the current study, such as conductor damage, missing or loose hardware, vegetation encroachment, and ice accumulation on insulators. Increasing intra-class diversity by collecting images across a wider range of tower geometries, seasonal conditions, and geographic regions can further improve model generalization and reduce false detections in visually ambiguous scenarios.

Additionally, a formally stratified per-region performance evaluation, computing mAP_50_ independently for each state or line segment, is considered an important direction for future research. Enabling this analysis requires retaining per-image segment identifiers throughout the annotation and dataset-splitting pipeline, which will be incorporated in future data collection cycles.

A systematic ablation study is also planned to quantify the individual contribution of the principal modules composing the proposed framework. Future work will investigate the impact of the offline asset grouping strategy on asset-level inspection consistency, as well as the contribution of the dedicated condition assessment models to the overall inspection performance. Such an analysis will require dedicated asset-level evaluation metrics beyond the image-level object detection measures adopted in this work.

The integration of complementary sensing modalities, particularly thermal and multispectral cameras, will also be investigated to enable the detection of anomalies that are not observable in standard RGB imagery. Thermal imaging, for instance, can reveal overheated connectors, resistive joints, and insulator hotspots that often precede electrical failures, while multispectral sensing may provide complementary information for identifying surface degradation and vegetation encroachment. Incorporating these modalities is expected to extend the proposed framework from structural inspection toward comprehensive electrical condition assessment.

Another promising research direction concerns the deployment of the proposed framework on edge-AI platforms embedded in UAVs. Recent advances in lightweight neural networks and dedicated AI accelerators make onboard inference increasingly feasible, allowing preliminary defect identification during flight while reducing communication and post-processing requirements. Such capabilities could support adaptive inspection missions, in which the UAV autonomously acquires additional images whenever potential anomalies are detected, moving toward real-time decision support.

Furthermore, a controlled benchmark involving different detection architectures, including RT-DETR, EfficientDet, Faster R-CNN [30], and recent YOLO releases (YOLOv8, YOLOv10, and YOLOv11) [13,14,15], trained and evaluated on the same multi-regional dataset under identical conditions, is considered an important future research direction. Such an evaluation would provide quantitative evidence regarding the trade-offs between single-stage, two-stage, and transformer-based detectors for large-scale UAV-based power line inspection.

The adoption of Vision Foundation Models (VFMs) and multimodal learning architectures also represents a promising avenue for future work. Their ability to exploit large-scale pretraining may improve robustness in scenarios with limited annotated data while facilitating adaptation to new asset categories and inspection environments.

Finally, future developments will investigate integrating Vision-Language Models (VLMs) with the proposed inspection pipeline to provide semantic interpretation of detected anomalies, automated generation of technical inspection reports, and natural-language interaction with maintenance personnel. In addition, combining inspection outputs with historical maintenance records and asset degradation models may enable predictive maintenance strategies that estimate asset health trends and support risk-informed maintenance planning. 

## Figures and Tables

**Table 1:** Representative studies on UAV-based inspection of power infrastructure.

Reference	Target	Method	Main Contribution
Liu and Liu [9]	Transmission lines	Deep Learning review	Overview of recent AI approaches and future research directions.
Mendu and Mbuli [10]	General inspection	Review	Survey of UAV technologies and sensing modalities for power infrastructure inspection.
İnce [11]	Smart grid	AI survey	Review of machine learning applications in power-system monitoring.
Liang et al. [1]	Insulators	CNN	Automatic detection of insulators from UAV images.
Maduako et al. [2]	Components	Deep Learning	Recognition of power-line assets using computer vision.
Chen et al. [20]	Defects	YOLO	Improved defect detection under field conditions.
Kang and Kim [21]	Anomalies	Real-time DL	Low-latency framework for transmission-line inspection.
Liu et al. [18]	Tiny airborne objects	Detection + Tracking	Efficient UAV perception framework combining lightweight detection and temporal association for robust airborne object perception.
Fu et al. [19]	Infrared small targets	SAM-guided Prompt Learning	Foundation-model-based framework for infrared small target detection using self-prompt learning and contextual representations.
This work	Components + anomalies	YOLOv11-M + Offline grouping	End-to-end framework integrating autonomous UAV inspection, multiclass object detection, condition assessment, asset-level grouping, and large-scale field validation across six transmission and distribution lines.

**Table 2:** Dataset composition for the detection and classification models.

Task	Class	Total Annotations
Detection	Rufous-hornero nest	424
	Rust	3208
	Polymeric anchoring insulator	2653
	Pin-type insulator	2412
	Ceramic disc insulator	4367
	Crossarm	1278
	Glass disc insulator	19,230
	Double-glass disc insulator	2860
Classification	Polymeric insulator—Clean	3909
	Polymeric insulator—Dirty	897
	Pin attachment—Cable tie	441
	Pin attachment—Elastomeric ring	1049
	Pin attachment—Untied	415
	Pin attachment—Plastic side tie	264
	Ceramic insulator pin—Normal	2270
	Ceramic insulator pin—Damaged	229
Total images in the dataset		≈19,065

**Table 3:** Hyperparameters used in training the YOLO-based detection model.

Hyperparameter	Value
Input resolution	640 × 640
Batch size	32
Optimizer SGD with momentum	0.9
Learning rate SGD (initial)	0.01
Learning rate schedule	Linear decay
Weight decay	0.0005
Warmup epochs	3
Total epochs	900
Epochs patient	100
IoU threshold (NMS)	0.70
Confidence threshold	0.25
Data augmentation	Rotation, scaling, translating
Anchor configuration	Anchors free
Loss function	Box, cls, dfl

**Table 4:** Characteristics of the DLs and TLs considered in the system evaluation. The Brazilian states are presented by their abbreviations: BA (Bahia), MG (Minas Gerais), MT (Mato Grosso), PR (Paraná), and RN (Rio Grande do Norte).

ID	Start/End	State	Cables	V (kV)	Length (km)	Towers
TL–01	Janaúba Solar Plant Substation/Janaúba Substation	MG	3	500	20.6	48
TL–02	Renascença Wind Power Plant Substation/João Câmara Substation	RN	3	138	10.7	41
TL–03	Nova Guaporé Hydroelectric Plant Substation/Jauru Substation	MT	2	138	32.1	125
DL–01	Pedrinho I Hydroelectric Plant/Pitanga Substation	PR	3	69	46.3	711
DL–02	Alto Sertão Wind Turbines/Alto Sertão Wind Power Plant Substation	BA	3	34.5	78.9	1140
DL–03	Renascença Wind Turbines/Renascença Wind Power Plant Substation	RN	3	34.5	42.6	1900

**Table 5:** Environmental and structural characterization of the inspection lines, where D means the density of towers per kilometer (t/km).

ID	D	Biome/Terrain	Primary Visual Challenges
TL-01	2.3	Atlantic Forest/Mountainous	Fog, dense vegetation, complex background
TL-02	3.8	Caatinga/Flat coastal	High luminosity, arid uniform background
TL-03	3.9	Amazon transition/Tropical lowland	Tropical haze, humidity, canopy occlusion
DL-01	15.4	Atlantic Forest/Subtropical plains	Variable cloud cover, heterogeneous vegetation
DL-02	14.5	Caatinga/Semi-arid plateau	High-contrast glare, dust, sparse vegetation
DL-03	44.6	Caatinga/Flat	High luminosity, uniform background, dense pole network

**Table 6:** Metrics of the final result of the detection model validation.

Asset Type	Class	mAP_50_	Precision	Recall	FP	FN	TP	Total	Epochs
Rufous-hornero nest	–	0.9245	0.9328	0.8378	4	20	96	116	311
Rust	–	0.8945	0.8990	0.8603	74	38	370	408	601
Polymeric anchoring insulator	–	0.9645	0.9401	0.9258	162	163	2236	2399	364
Pin-type insulator	–	0.9788	0.9810	0.9127	28	37	444	481	304
Ceramic disc insulator	–	0.9935	0.9813	0.9802	44	14	1299	1313	301
Crossarm	–	0.9627	0.9364	0.9150	26	14	233	247	654
Insulators (multiclass detection)	Glass disc insulator	0.9817	0.9555	0.9648	228	68	3576	3644	518
Insulators (multiclass detection)	Double-glass disc insulator	0.9817	0.9555	0.9648	42	7	533	540	518

**Table 7:** Metrics of the final result of the classification model validation.

Condition (Epochs)		Accuracy	P	R	FP	FN	TP	Total
Polymeric anchoring	Clean insulator	0.97	0.97	0.99	20	6	776	782
insulator (436)	Dirty insulator	0.97	0.96	0.89	6	20	160	180
	Cable tie	0.92	0.86	0.96	13	3	82	85
Insulator pin	Elastomeric ring	0.92	0.95	0.96	10	8	193	201
pin attachment (84)	Untied	0.92	0.89	0.76	7	19	60	79
	Plastic side tie	0.92	0.96	0.96	2	2	49	51
Ceramic insulator	Damaged	0.98	0.86	0.95	6	2	37	39
pin (166)	Normal	0.98	0.99	0.99	2	6	454	460

**Table 8:** Comparison between conventional and UAV-based inspection methodologies.

Metric	Outsourced	Manual Team	Proposed
Cost (US$/km)	392.6	81.5	9.8
Human-hours/km	–	5.0	0.6
Cost reduction	–	88.0%	97.5% *
Productivity gain	–	–	8.3×

* Relative to outsourced inspections.

## Data Availability

The data used to support the findings of this study are available from the corresponding author upon request.
